# Energy and Capital Cost Reduction in Ester Transesterification Using an Optimized Reactive Distillation System with Prefractionation

**DOI:** 10.1021/acsomega.5c07614

**Published:** 2025-10-29

**Authors:** Carles Troyano Ferré, Ruben Cabello, Alvaro Risco, Alexandra Elena Plesu Popescu, Jordi Bonet

**Affiliations:** Faculty of Chemistry, Department of Chemical Engineering and Analytical Chemistry, 16724 University of Barcelona, Martí i Franquès Street 1, Sixth Floor, 08028 Barcelona, Spain

## Abstract

Reactive distillation is a highly efficient process, but its application presents significant challenges in mixtures that have azeotropes, such as in the transesterification of methyl acetate (MeAc) and methanol (MeOH) with *n*-butanol (BuOH). This paper proposes a reactive distillation with a prefractionation column (RDPFC) system to valorize the waste stream from the poly­(vinyl alcohol) industry (30.7 wt % MeAc and 69.3 wt % MeOH) to recover high-purity MeOH and valuable *n*-butyl acetate (BuAc). The process was modeled and optimized in Aspen Plus version 12.1, with the objective of minimizing the total annual cost (TAC). The proposed system incorporates a prefractionation column (PFC) consisting of 26 stages working under vacuum conditions (0.6 atm) alongside a reactive column (RC) with 38 stages operating at high pressures (8 atm) to improve the conversion of the reaction. The RDPFC process attains a MeAc conversion rate of 99.2 mol %, yielding products that align with commercial specifications, including over 99.85 wt % MeOH and more than 99.5 wt % BuAc. A comparative analysis reveals that the RDPFC configuration utilizes 2.56 GJ/tBuAc, showcasing a significant 67% reduction in energy consumption when compared to the traditional reactive and extractive distillation (RED) process. The removal of the entrainer and its corresponding recovery unit primarily accounts for this improvement. This study suggests that the RDPFC system could be a highly efficient and economically attractive solution for valuing waste streams in the poly­(vinyl alcohol) industry.

## Graphical abstract

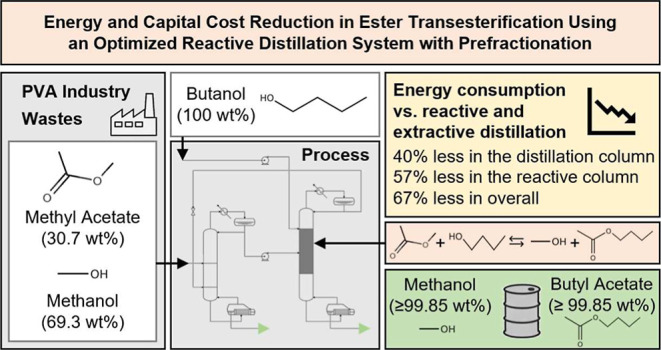

## 1 Introduction

As a technology that matured over hundreds of years, distillation continues to be a primary industrial method for separating liquid mixtures into their pure components at a large scale.^1^ The separation is the result of a progressive distribution of species between the vapor and liquid phases along the column, which are created by heat transfer. Although the separation can be performed without adding chemical compounds that would require more separation units—like in methods relying on mass separation agents—distillation remains an energy-intensive technology that poses challenges for sustainable chemical processing.^2^ In the United States, for instance, approximately 91 GW are consumed each year by distillation columns,^3^ which account for more than the total energy generated by nuclear plants in this country over the year 2022.^4^ Consequently, over the decades, studies have focused on reducing energy requirements of this process, and new techniques and configurations have been proposed to enhance its energy efficiency.^1^ Some of these improvements involve integrating distillation sequences energetically^5^ or replacing the existing systems with intensified designs such as thermally coupled prefractionators^6^ or dividing wall columns.^7^

Industrial chemical processes can be improved further by integrating the separation operation with the chemical reaction. Reactive distillation is an enhanced technique that combines the reaction and distillation operations in a single unit. Hence, the application of this integrated process results in considerable savings in capital investments and energy costs in comparison to the conventional reaction and separation sequence. Moreover, reactive distillation offers significant benefits for equilibrium reactions by enabling the continuous removal of products, facilitating complete conversion, and overcoming azeotropes through changes in the liquid phase composition. However, there are instances in which achieving the desired composition in product streams is either economically inviable or simply impossible due to the close-boiling nature of the mixture or the formation of azeotropes, respectively. In these situations, reactive distillation is combined with additional separation processes, such as other enhanced distillation technologies—e.g., extractive and pressure-swing distillation—or pervaporation, to reach the required purity.^8^

### 1.1 Methyl Acetate Transesterification Case Study

Methyl acetate transesterification is an example of a system in which hybrid processes have been studied to enhance its production. The alcoholysis of methyl acetate (MeAc) produces an acetate ester with the alcohol’s organyl group and methanol (MeOH), which forms a minimum-boiling azeotrope with MeAc. This reaction, typically catalyzed by acids, has an equilibrium constant near one, resulting in a relatively low conversion with a single contact between the reactants.^9^ Therefore, reactive distillation combined with extractive, pressure-swing, dividing wall column distillation, or pervaporation has been proposed to separate this azeotrope, recycle unreacted MeAc, and enhance the conversion.

#### 1.1.1 Reactive and Extractive Distillation

In this process (Figure 1), a solvent is used to overcome the azeotrope and improve the contact between the reactants. One of the advantages of this process is its ability to adjust reactants’ and products’ relative volatilities with the entrainer. The most suitable entrainers for the MeAc–MeOH mixture are alkylbenzenes and alkanes. If factors such as safety, cost, or physical properties are considered, *o*-xylene has been proved to be the best entrainer.^10^ In the past decade, the usage of ionic liquids as entrainers has also been thoroughly researched. The most relevant property of these compounds is having a negligible vapor pressure. Hence, they can easily be separated from volatile chemicals by ordinary distillation and thus reduce energy consumption. Another significant property is their acidity, which enables their use as catalysts.^11^ Consequently, specific ionic liquids can be used as both entrainers and catalysts in this process. Nonetheless, extractive distillation processes share the drawbacks of operations based on the use of mass separation agents, such as the requirement of an additional unit to recover the solvent for recycling or an entrainer makeup.^12^

**Figure 1:**
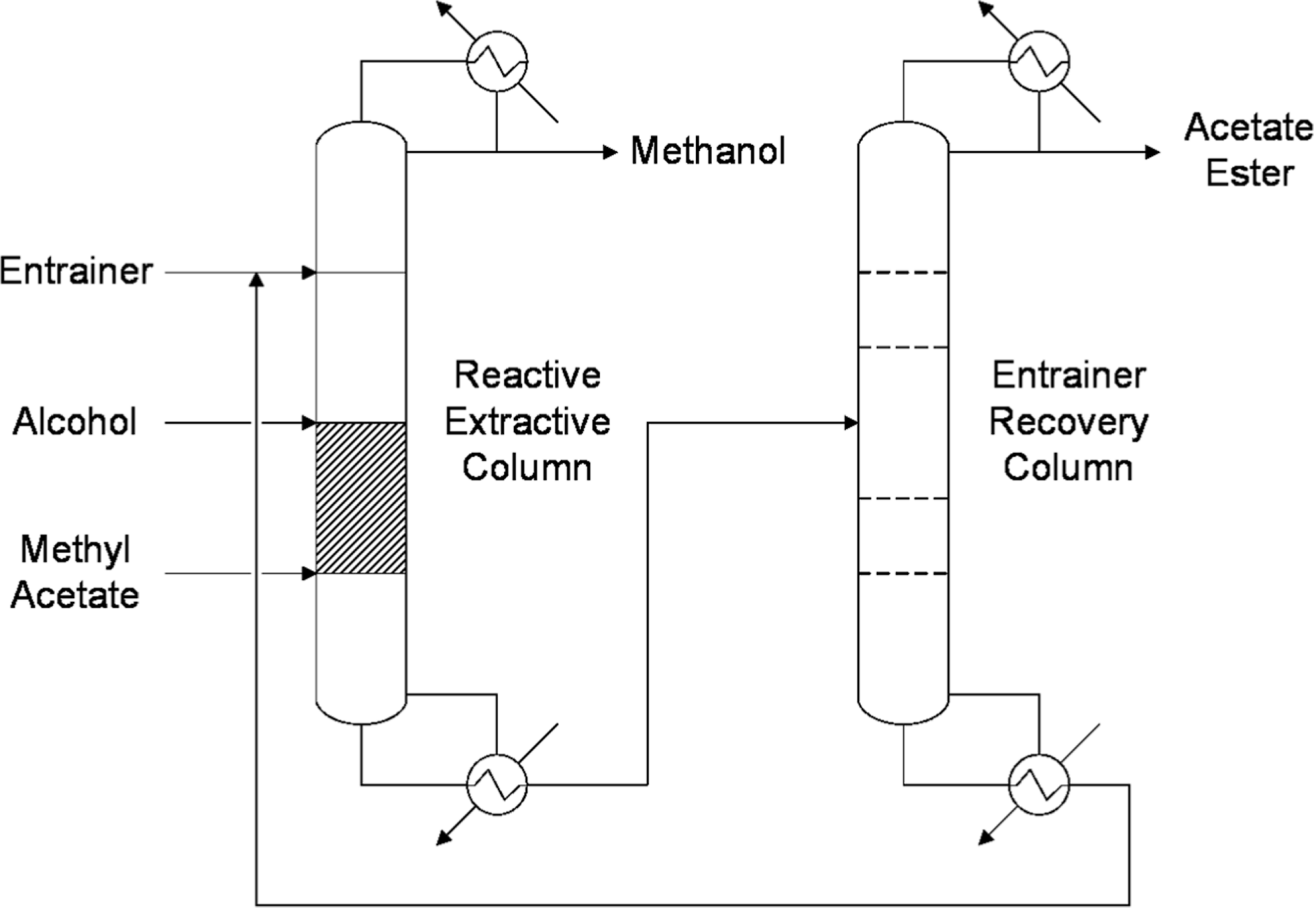
Reactive and extractive distillation process flow diagram.

#### 1.1.2 Reactive Pressure-Swing Distillation

Pressure-swing distillation is a technique in which an azeotrope is separated by using two columns operating at different pressures, exploiting the variation of its composition with this operating variable without the dependence on additional compounds. However, this technology is only feasible when the azeotropic composition undergoes a variation higher than five percent with a pressure change that does not exceed 10 atm.^13^ MeAc–MeOH azeotropic composition varies more than 8% when pressure is increased to 2 atm from atmospheric pressure; thus, overcoming the thermodynamic limitation using this technique is practicable (Figure 2). Furthermore, raising the reactive column operating pressure increases transesterification conversion, which involves a reduction of the reboiler duty required to achieve purity specifications.^14^

**Figure 2:**
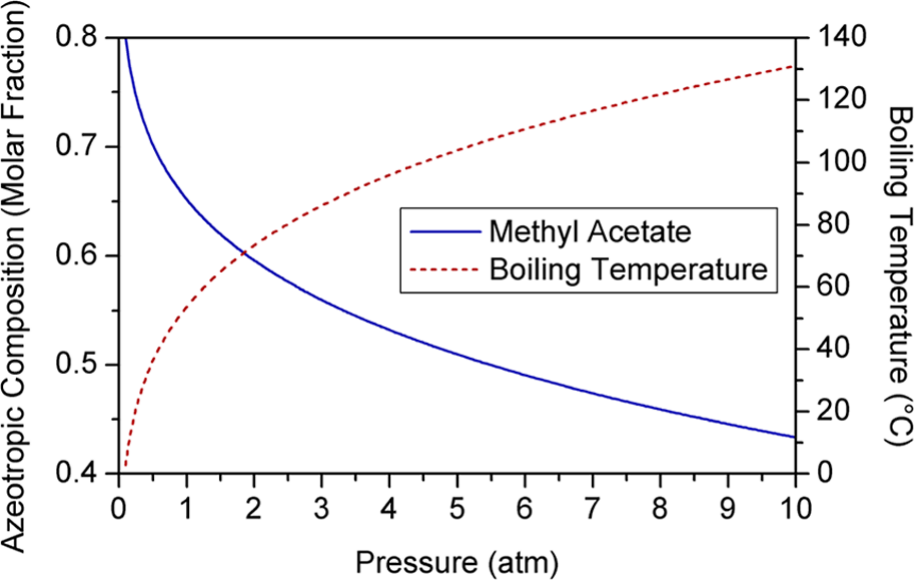
Impact of pressure on MeAc–MeOH azeotropic composition.

In this hybrid process (Figure 3), reactants are introduced at both ends of the reaction zone in the high-pressure column; the ester produced during the reaction is extracted from the bottom with the desired purity, and a mixture of MeOH and unreacted MeAc is collected as the overhead product. The reactive column distillate is then introduced into the separation column that operates at atmospheric pressure, where MeOH is purified and collected from the bottom and the azeotropic mixture is recycled to the high-pressure column. Since the temperature of the reactive column is higher than the low-pressure column bottoms, the high-pressure column condenser and separation column reboiler can be integrated energetically to enhance efficiency.^14,15^ This integration is just provided if the temperature difference is higher than the optimal minimum temperature approach—usually 10 °C.^16^

**Figure 3:**
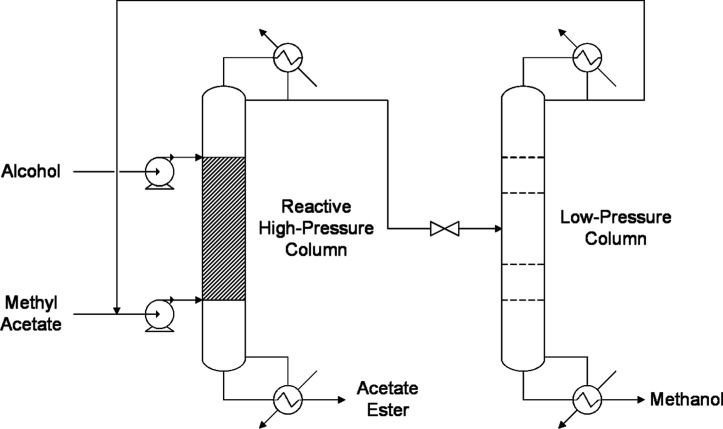
Reactive pressure-swing distillation process flow diagram.

#### 1.1.3 Reactive Dividing Wall Column Distillation

Dividing wall column (DWC) distillation is a process that fully integrates the columns of a conventional distillation sequence into a single shell. This design allows obtaining the target products, and even highly concentrated side products, consuming up to 45% less energy than ordinary separation arrangements and reducing investment costs considerably.^8^ Suo et al. (2017) studied the integration of the reactive column and the prefractionation column for the alcoholysis of MeAc, where stripping sections are partitioned by the wall (Figure 4).^17^ MeAc and *n*-butanol (BuOH) are introduced into the reactive part of the column, and the generated *n*-butyl acetate (BuAc) is collected at the bottom. The liquid mixture of MeOH and unreacted MeAc flows from the rectifying section of the DWC’s reactive side to its prefractionation part, in which the generated MeOH is separated from the MeAc–MeOH azeotrope in the stripping section of this zone as a side product. The azeotropic mixture is then recycled to the reactive side of the DWC and mixed with the fresh MeAc. According to the authors, this intensified process involves 26% energy savings compared with the conventional reactive distillation process.^17^ However, despite significant savings, DWC distillation has some disadvantages. A DWC process has more stages than a nonintensified system; consequently, the pressure drop within the unit is higher, which increases the temperature gap between the reboiler and condenser and may lead to utilities-related problems.^8^ Additionally, operating pressure is the same for both parts of the column; therefore, although a rise in pressure results in an increase in the conversion on the reactive side, it also entails an increase in the MeOH azeotropic composition on the fractionation side. Hence, larger flow rates of distillate must be recycled to achieve the specifications, and more energy will ultimately be required by the reboiler.

**Figure 4:**
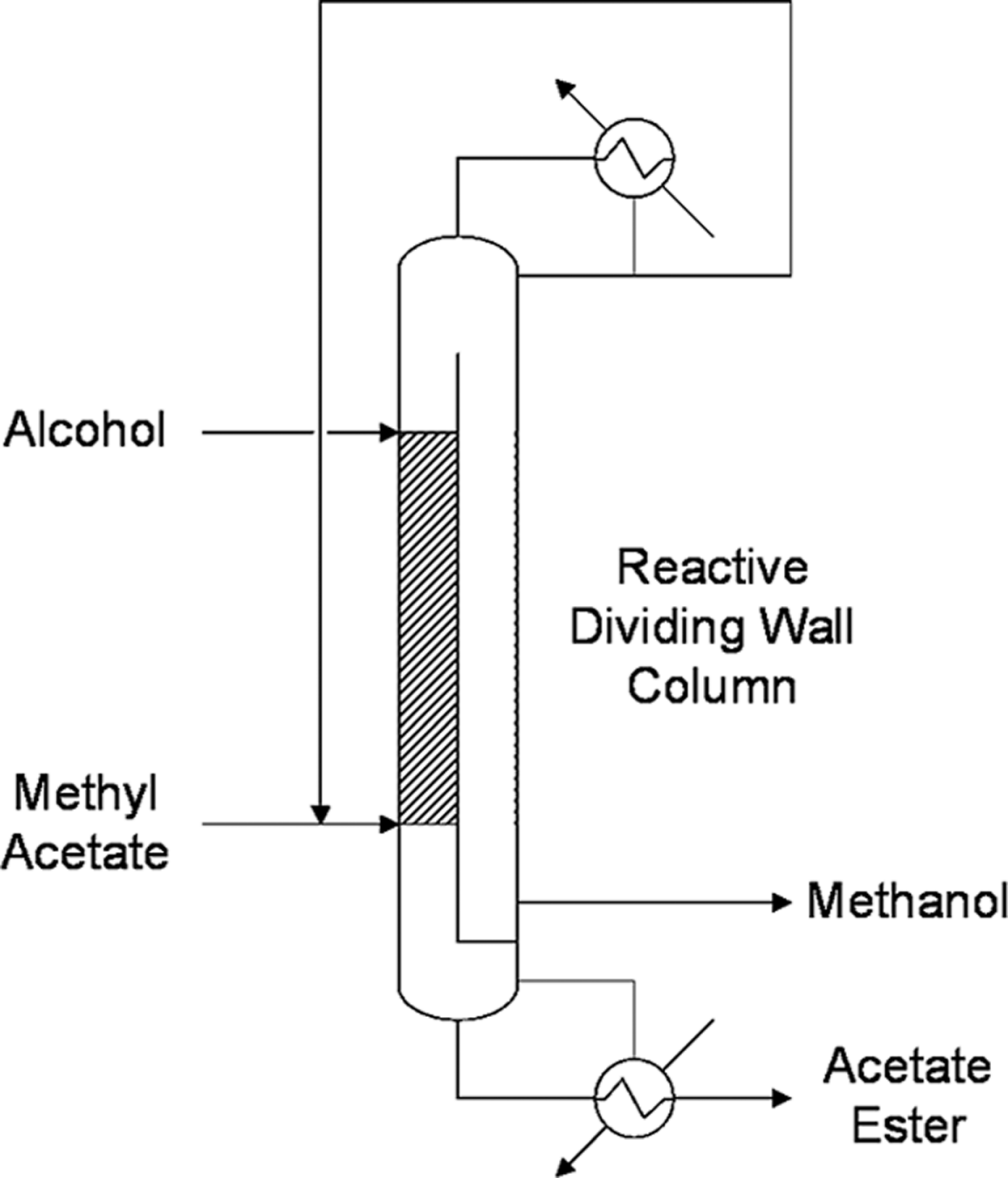
Reactive dividing wall column distillation process flow diagram.

#### 1.1.4 Reactive Distillation with Pervaporation

Pervaporation is a separation technique that uses a physical barrier, a membrane, to overcome thermodynamic limitations. In this process, the liquid mixture is kept at a pressure high enough to remain in its liquid state. Components with a higher permeability pass through the membrane and are continuously removed as vapors, known as the permeate. Meanwhile, species with lower permeabilities remain in the liquid phase and are collected as retentates. For the MeAc–MeOH azeotrope, when low-selectivity membranes are used, the permeate stream is mainly constituted of MeOH. However, large membrane areas are needed to attain a highly concentrated retentate since the concentration gradient is the driving force for this process, and a distillation column is needed to separate further MeOH from MeAc (Figure 5a). High concentrations of components on both sides of the barrier are achievable by implementing a high-selectivity membrane. Reactive distillation combined with high selectivity pervaporation (Figure 5b) allows for the achievement of high purities of both the acetate ester and MeOH without the need for a second distillation unit. This configuration significantly reduces energy and equipment costs in comparison with reactive distillation with low-selectivity pervaporation.^18^ Nevertheless, using membranes in the industry involves drawbacks that are ultimately associated with their lifetime, which directly affect operating costs. Some of these disadvantages are the need for long-life membranes, the requirement of modules of high surface areas, and cleanup operations to prevent membrane deterioration and degradation due to caking, plugging, and fouling.^12^

**Figure 5:**
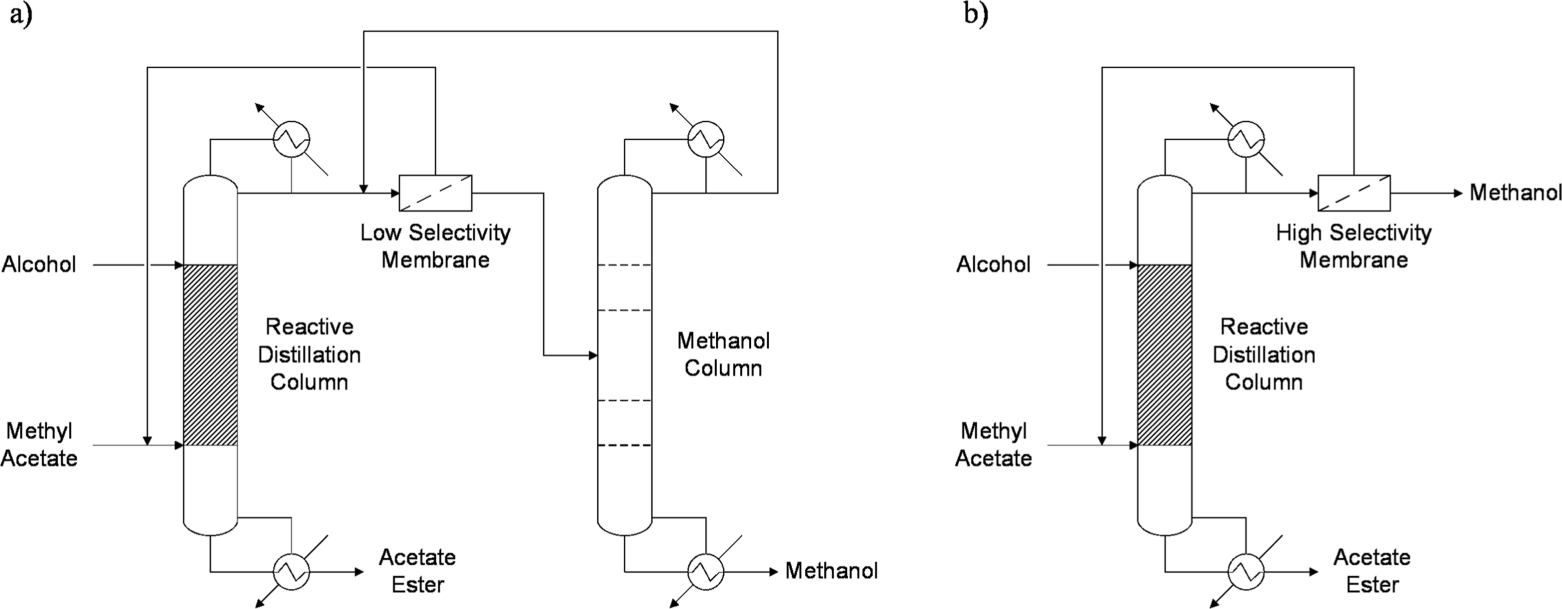
Reactive distillation with (a) low-selectivity and (b) high-selectivity pervaporation.

### 1.2 Poly­(vinyl alcohol) Synthesis

MeAc is mainly obtained from acetic acid synthesis,^19^ but it is also generated in large amounts as a byproduct in several other industries, such as the poly­(vinyl alcohol) (PVA) process. PVA is a biodegradable, water-soluble, and nontoxic polymer whose relevance has risen in the last few decades due to the increasing concern over the harmful effects of conventional petroleum-based plastics on the environment.^20,21^ PVA is commercially manufactured in both batch and continuous processes by poly­(vinyl acetate) transesterification with MeOH because its monomer is nonexistent and, thus, cannot be synthesized via polymerization. The polymer is then washed with MeOH and separated from the generated MeAc, the MeOH used in the process, and other impurities by using filters and dryers.^20,22^ In the manufacturing process, 1.68 kg of MeAc is produced per kg of PVA; however, the ester contained in the resulting filtrate is diluted with MeOH.^23^ Consequently, MeAc cannot be separated from MeOH by conventional distillation since it would form the above-mentioned azeotrope with the alcohol.

Currently, to recover MeOH and reuse it in polymer synthesis, the MeAc–MeOH azeotrope is processed to transform the ester into acetic acid and MeOH by hydrolysis in an extractive distillation column. The resulting products are subsequently separated and purified by using extraction or azeotropic distillation. Nonetheless, the implementation of such technologies makes the PVA process highly capital-intensive,^20^ which results in a significant reduction in net income considering the low value of acetic acid in the market. Hence, using a hybrid and intensified distillation technique to produce more valuable chemicals from MeAc is an attractive alternative for this industry.

### 1.3 Prefractionation Column Integration

According to chemical engineering heuristics,^16,24^ chemicals whose amount within the system is the largest should be removed first using cheap separation methods to decrease the volume of the subsequent units and thus reduce capital costs. Additionally, the separation of complex mixtures should be postponed until the end because their associated costs tend to be higher. Taking these rules into account, the MeAc–MeOH azeotrope must be processed in the last unit of the sequence, and the MeOH not taking part in this binary mixture must be separated first using a preconcentration column. MeAc is withdrawn as distillate and can then be converted into BuAc, an ester commercially used as a solvent^19^ whose price is higher than acetic acid’s or the MeAc value itself, by reactive distillation.

Although the reactive DWC technique offers important savings compared to the other methods described in Section 1.1, it must be noted that, for the PVA residual stream case study, the MeAc used as feedstock is fairly diluted. Hence, the number of stages of the reactive zone and the energy consumed by the unit will be much larger than the results reported by Suo et al. (2017)^17^ because MeAc concentration and thus its conversion will be rather smaller. Therefore, considering that the operating pressure of the DWC reaction section cannot be raised to increase the reaction conversion due to the inconveniences it entails (Section 1.1.3), using a separate reactive column is preferred. The binary mixture obtained in the overhead product of the reactive column (RC) can subsequently be separated by using a low-pressure column exploiting the effect of pressure variation, as explained in Section 1.1.2. However, since this stream is only comprised of MeAc and MeOH, these species can be separated by feeding them into the prefractionation column, instead of using an independent column, resulting in a further decrease in costs. The combination of the preconcentration column with a unit of an enhanced distillation sequence was previously studied and optimized for extractive distillation,^25^ but to our knowledge, its application to a reactive distillation system with azeotropes has not been researched.

The treatment of poly­(vinyl alcohol) (PVA)’s residual stream, which is made up of MeAc and MeOH, necessitates the modeling, analysis, and optimization of a reactive distillation process that incorporates a prefractionation column. The goal is to increase the value of the residual stream while minimizing capital and energy expenditures in the overall process. The feedstock preconcentrator and the unit that processes the RC’s overhead product are both used for MeOH separation and are combined in one column because their feed compositions are similar, which saves energy and lowers capital costs. The RC converts azeotropic MeAc and BuOH into MeOH and BuAc by transesterification. Section 2 discusses the reaction kinetics, thermodynamic models, and sensitivity analysis methodologies used to simulate and optimize the distillation sequence that produces commercially acceptable products. Design parameters such as the number of reactive stages, reflux ratio, and operating pressure are investigated (Section 3) to increase energy efficiency and demonstrate the advantages of this hybrid distillation process. While the application of a preconcentration column followed by a reactive-extractive distillation has been explored for valorizing an industrial waste stream,^26^ its application to a reactive distillation with prefractionation remains a key research gap. The proposed Reactive Distillation with a Prefractionation Column (RDPFC) system directly addresses this gap. Unlike fully integrated systems, such as the reactive dividing wall column (DWC), where reaction and separation conditions are coupled and cannot be independently optimized, our proposed configuration decouples these operations. This decoupling may offer a flexible and highly efficient solution.

## 2 Methodology

The schematic flow diagram for the process proposed for the separation and synthesis of BuAc and MeOH is represented in Figure 6. This reactive distillation with a prefractionation column system is modeled and optimized with Aspen Plus version 12.1 by using modules based on the equilibrium-stage concept and minimizing the energy consumed by these units.

**Figure 6:**
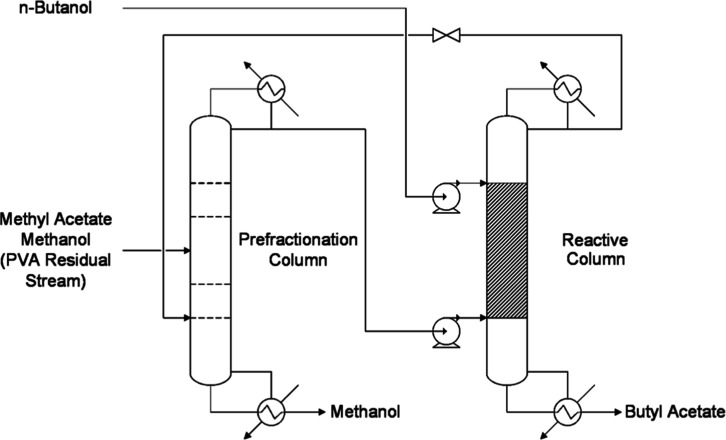
Schematic process flow diagram.

### 2.1 Reaction Kinetics and Thermodynamic Model

The alcoholysis of MeAc with BuOH was studied by Jiménez et al. (2002) in the presence of the ion-exchange resin Amberlyst 15 (Table 1).^10^ Transesterification is a reversible second-order reaction, and its kinetics is usually expressed using the pseudohomogeneous model (eq 1) because it occurs in the liquid phase and mass transfer resistance can be neglected.^27^ The variation of pre-exponential factors with temperature is described by the Arrhenius equation (eq 2).

1 $$r=k^{+}c_{MeAc}c_{BuOH}-k^{-}c_{BuAc}c_{MeOH}$$

2 $$k=k_{0}\exp\left(-\frac{E_{a}}{RT}\right)$$

where *r* is the reaction rate, *k* ^+^ and *k* ^–^ are forward and backward reactions rate constants, respectively, *k*_0_ is the pre-exponential factor, and *E*_a_ is the activation energy.

**Table 1:** Kinetic Parameters for MeAc Transesterification with BuOH^10^

	pre-exponential factor	activation energy
	m^3^ kmol^–1^ s^–1^ kg_cat_^–1^	kJ mol^–1^
forward reaction	3.36 × 10^9^	71.96
backward reaction	4.73 × 10^9^	72.67

The transesterification rate depends on the amount of catalyst used in the reactive column; consequently, it affects the energy requirements of the unit. Therefore, a large amount of catalyst is used to avoid computing the effect of the catalyst loading in the analysis of other design variables since factors such as catalyst lifetime or price must be considered to select its appropriate amount. This adjustment also involves achieving a conversion close to equilibrium in each reactive stage.

The simulation was performed using the nonrandom two-liquid (NRTL) model, which considers liquid phase nonidealities using activity coefficients, with the ideal gas vapor equation of state. Built-in binary parameters (Table 2) were developed by AspenTech using the Dortmund Data Bank.^28^ The NRTL model is adequate for moderate operating conditions, including pressures under 10 bar.^29^

**Table 2:** Binary Interaction Parameters of the NRTL Model^28^

component *i*	component *j*	*A*_*ij*_	*A*_*ji*_	*B*_*ij*_	*B*_*ji*_	α_*ij*_
methyl acetate	methanol	0.0	0.0	234.866	130.505	0.3
methyl acetate	*n*-butyl acetate	–10.0572	7.7169	3508.99	–2651.09	0.3
methyl acetate	*n*-butanol	7.4512	–6.8907	–2197.74	2244.39	0.3
methanol	*n*-butyl acetate	0.0	0.0	140.976	234.499	0.3
methanol	*n*-butanol	2.22	–1.5165	–337.712	242.624	0.3
n-butyl acetate	*n*-butanol	–3.0296	1.7609	1122.14	–429.639	0.3

### 2.2 Process Modeling and Optimization

The feed into the prefractionation column (PFC) has a flow rate of 100 kmol/h and consists of a binary mixture of MeAc and MeOH (30.7 wt % MeAc).^30^ Columns are simulated with RadFrac modules using total condensers and assuming zero pressure drop along these units. The PFC is initially set up with a high number of stages in the rectifying and stripping sections and a large reflux ratio. Similar to the preconcentrator, the reactive column (RC) initially contains a sufficient number of trays in both the rectifying and stripping zones to achieve desired separation of overhead and bottom products, thereby ensuring that its impact on the reboiler duty remains negligible regardless of the reflux ratio. All stages of the RC are specified as reactive by indicating the reaction kinetics and using 50 kg of catalyst in each tray. PFC’s distillate is introduced into the last reactive stage of the RC. A stream of pure BuOH is fed into the first stage of the reaction, where it subsequently reacts with the ester involved in the azeotrope. BuOH flow rate is forced to guarantee a BuOH/MeAc molar proportion of 1:1. Azeotrope and BuOH pressures are increased to the one specified in the RC by using pumps. The RC’s overhead product, mainly composed of MeOH purified in the prefractionator, undergoes an adiabatic flash when it is fed into the PFC.

Product streams’ purities are set to meet commercial specifications (Table 3) by automatically adjusting the required reflux ratios with the Design Specifications tool included in RadFrac units. Therefore, the design factor used to optimize the energy consumed by columns is the reflux ratio (RR) since reboiler (*Q*_R_) and condenser (*Q*_C_) duties themselves are not good design variables—specifying them might result in unrealizable conditions^12^—, but they strongly depend on this reflux ratio. Operating and capital costs, estimated with the built-in Aspen Process Economic Analyzer, are used to determine the optimal column configurations. Additionally, the minimum reflux ratio (RR_min_) of the prefractionator, calculated when the desired separation is attained with a significantly high number of theoretical stages, is employed to confirm that the optimum design complies with the optimal-reflux-to-minimum-reflux ratios heuristic 1.05 < RR/RR_min_ < 1.50.^12,24^ Due to the integration of the reaction section in the RC, applying the shortcut method to this unit might lead to inaccurate results. Design factors are optimized by running the Sensitivity tool and analyzing its effect on reboiler duties, which are affected by variation of the adjusted reflux ratio, as described above.

**Table 3:** Methanol and *n*-Butyl Acetate Purity Specifications^31,32^

		methanol	isobutyl acetate
purity on dry basis	wt % min	99.85	99.5
water	wt % max	0.10	0.05
acetone	mg/kg max	30	N/A
ethanol	mg/kg max	50	N/A
*n*-butanol	wt % max	N/A	0.5
acidity as acetic acid	mg/kg max	30	100

In summary, a sequential, iterative optimization was carried out using Aspen Plus v12.1. Parametric analyses with the Sensitivity tool varied key design variables—such as stage numbers, feed locations, and pressures. For each case, reflux ratios were adjusted via Design Specifications to meet product purity targets (Table 3). The impact on reboiler duties and the total annual cost (TAC) was assessed to identify optimal conditions. Capital and operating costs were estimated using an Aspen Process Economic Analyzer, ensuring a balanced design between investment and energy use.

## 3 Results and Discussion

The section includes the results obtained by simulating and optimizing the proposed reactive distillation system with a prefractional column (RDPFC). The influence of design factors, such as the number of reactive stages, reflux ratio, and operating pressure, is analyzed. Subsequently, a detailed optimization for both the prefractionation column and the reactive column is presented. Finally, the entire RDPFC process is reviewed, looking at its limitations and showing the cost and energy benefits of the new design.

### 3.1 Sensitivity Analysis of Design Factors

#### 3.1.1 Impact of Number of Reactive Stages

The MeAc conversion for a specific reflux ratio rises progressively as the number of reactive trays between feed stages increases (Figure 7). The interaction between fresh MeAc and BuOH in the same reactive stage leads to a conversion similar to that obtained in a conventional reactor. Since a large catalyst loading is used, equilibrium conversion is reached in each stage. Due to the significant difference in volatility of reactants, the introduction of feed streams into the same reactive tray results in unreacted MeAc and BuOH being predominantly transferred to upper and lower stages, respectively, in accordance with their physical properties. Since the concentration of one of the reactants decreases considerably, conversions yielded by the stages located above and below the feed stage are negligible, and thus, adding a catalyst to these trays is unnecessary. Feeding the heavier component at a higher stage in relation to the more volatile species creates a zone where the countercurrent flow of feedstock increases the reactant concentration, facilitating the generation of products.

**Figure 7:**
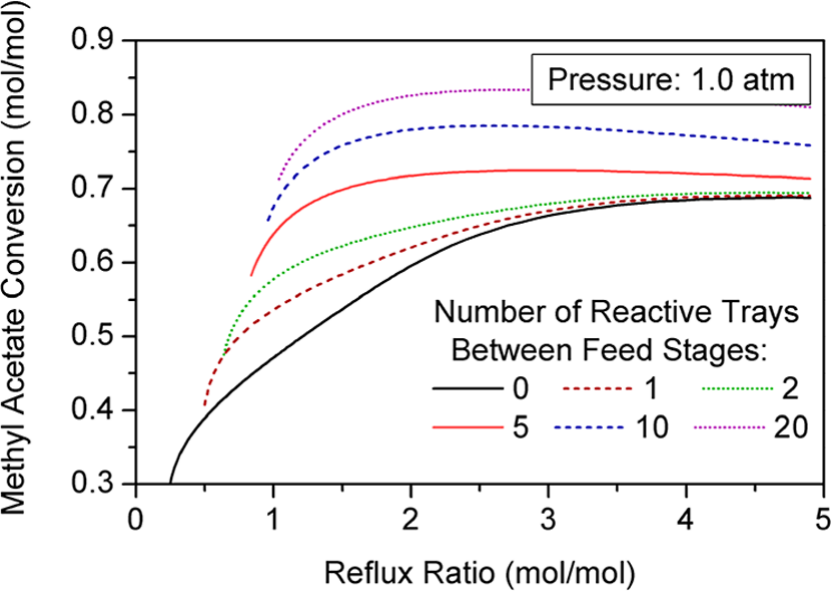
Effect of number of reactive trays between feed stages on conversion.

The stages in which larger amounts of BuAc are produced are feed stages because of the higher concentrations of MeAc and BuOH, and although the conversion attained by these stages decreases as the gap between feed streams increases (Figure 8), the overall yield of the unit is enhanced as shown in Figure 7.

**Figure 8:**
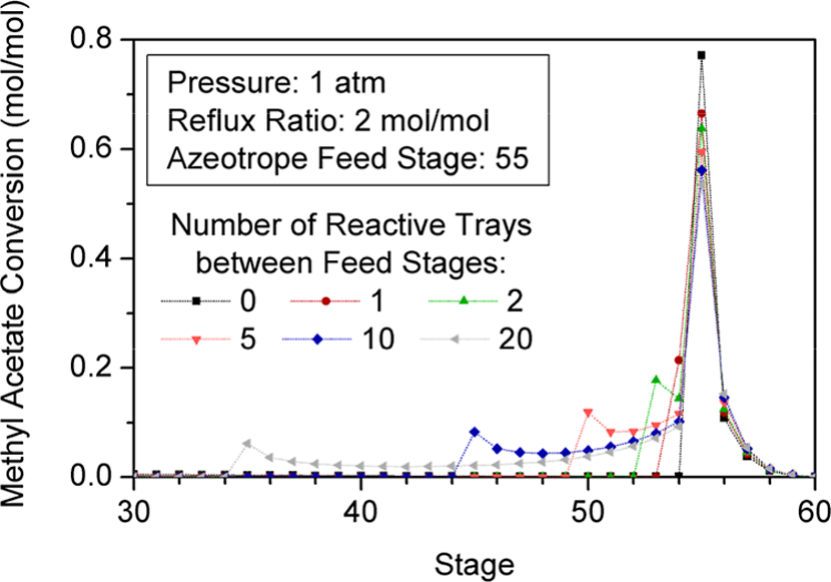
Reaction profile for different numbers of reactive stages between feed streams.

The rise of conversion with the number of reactive stages becomes gradually lower as the interval increases, tracking the curve of a function with a horizontal asymptote corresponding to 100% conversion (Figure 9). Hence, a huge gap between feed stages and thus a considerably large number of stages are required to obtain highly purified products.

**Figure 9:**
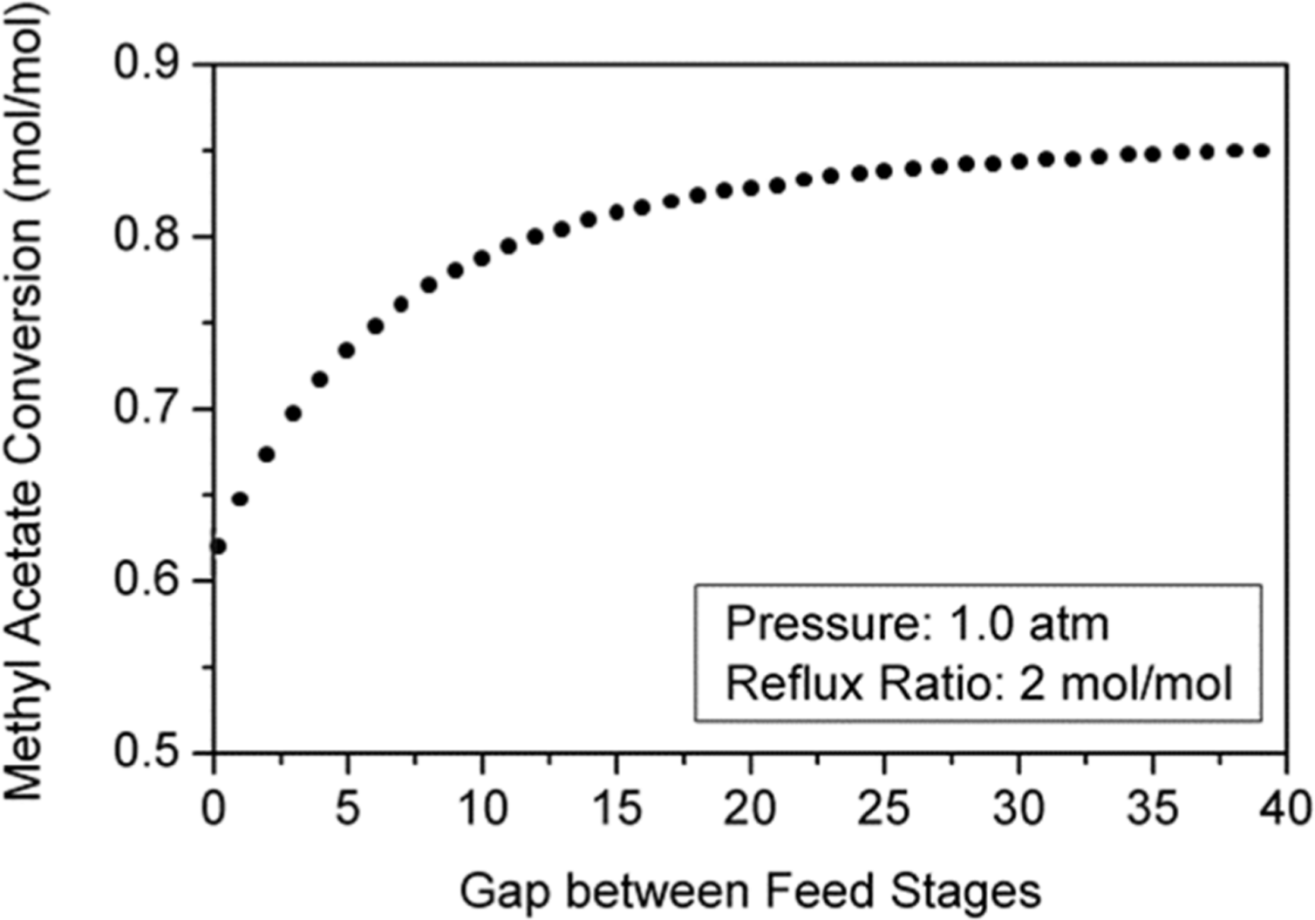
Variation of methyl acetate conversion with the interval between feed stages.

#### 3.1.2 Impact of Reflux Ratio

In conventional distillation columns, an increase in the reflux ratio results in a greater degree of separation of the components. Nonetheless, when a reaction takes place in this unit, the reflux ratio promotes other factors that also have an impact on the column performance. Figure 7 shows that raising the reflux ratio increases the conversion of reactants. However, once the maximum conversion is reached for a given number of reactive stages, a further rise of this design factor leads to a gradual decrease of the conversion.^33^ Figures 10 and 11 represent the impact of reflux ratios on composition and reaction profiles.

**Figure 10:**
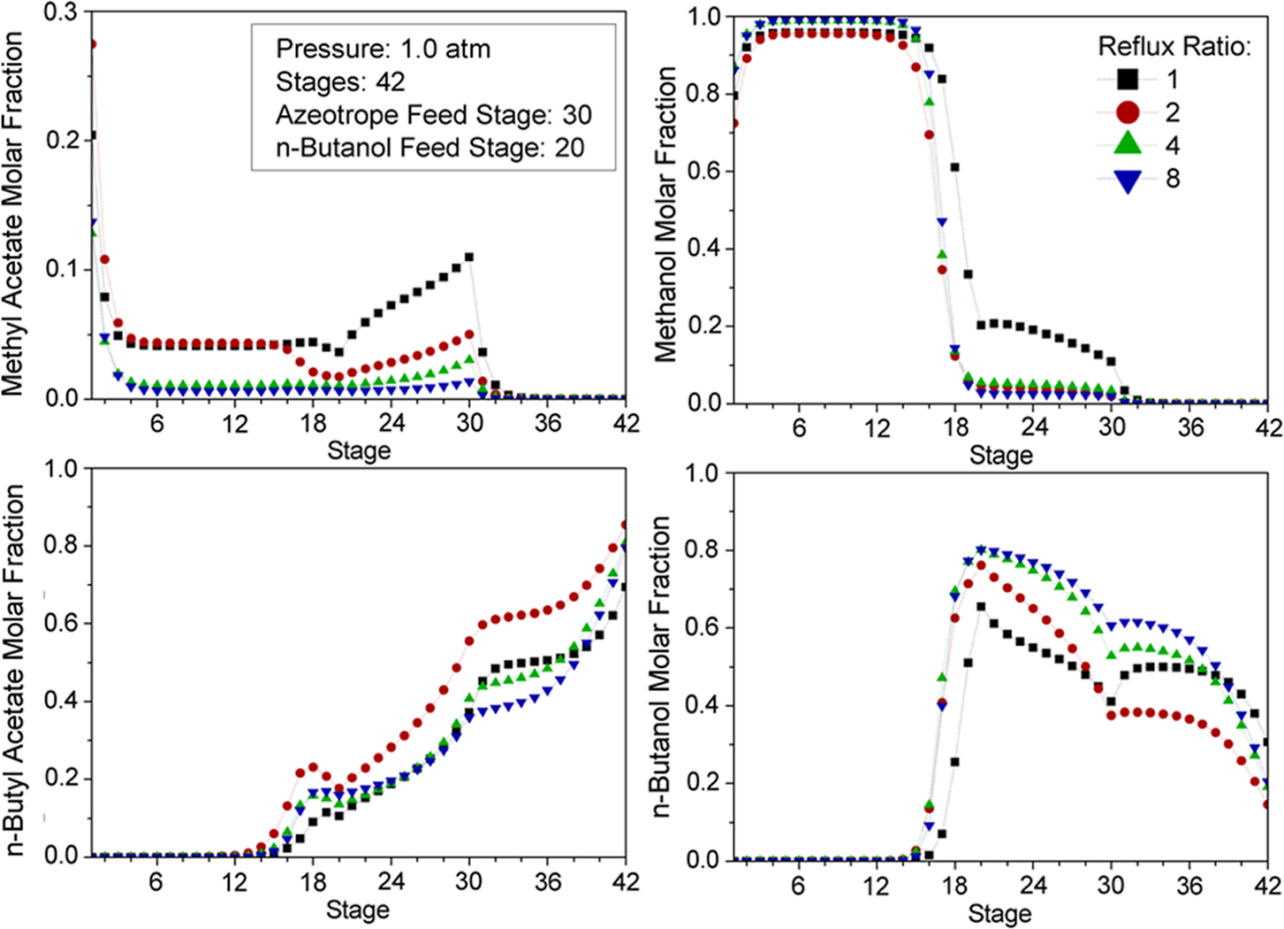
Effect of reflux ratio on the RC composition profile.

**Figure 11:**
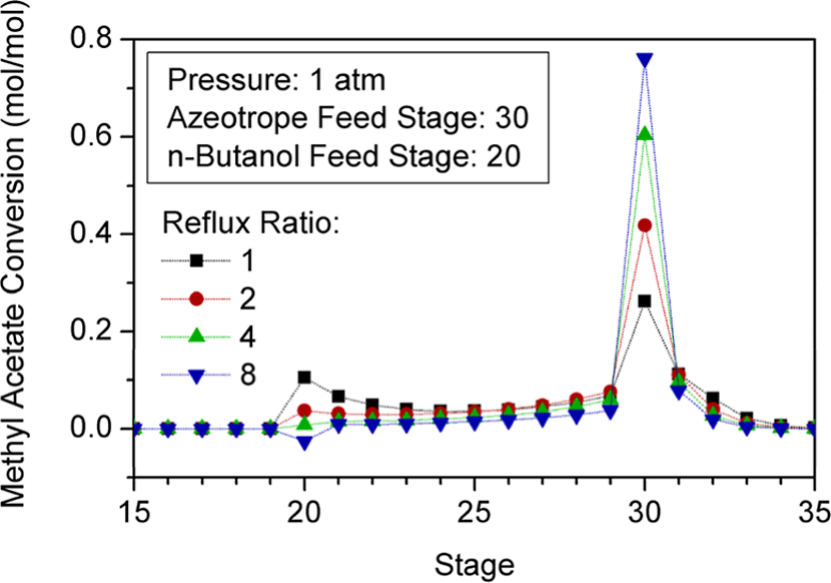
Effect of reflux ratio on the RC reaction profile.

Higher reflux ratios involve recirculating larger amounts of unreacted MeAc and, thus, entail a higher generation of products. Nevertheless, as the composition of MeAc along the column decreases (RR > 2 mol/mol), the concentration of BuOH in the liquid phase becomes gradually higher. Consequently, the fraction of BuOH in the bottom product (Figure 10: stage 42) increases with the reflux ratio, which naturally results in a lower purity of BuAc. Furthermore, a considerable rise of reflux ratio involves higher concentrations of MeOH in the upper stages of the reaction zone, due to its higher volatility, resulting in a progressive decrease of BuAc generation. When the concentration of products is substantially higher than that of the reactants, catalysts promote the inverse reaction instead. This conversion reduction is clearly shown in Figure 11 at stage 20, where the 8 mol/mol reflux ratio exhibits less conversion than lower reflux ratio values.

#### 3.1.3 Impact of Pressure

Conversion of reactants can be enhanced further by raising the operating pressure (Figure 12). The increase in MeAc conversion with pressure follows the same asymptotical trend shown in Figure 9. Therefore, achieving product specifications is possible by increasing both the number of reactive stages and the operating pressure. Since the conversion yielded by columns with few reactive trays is lower than that attained with larger reaction sections, the operating pressure of the former configurations must be higher to reach the desired composition in product streams.

**Figure 12:**
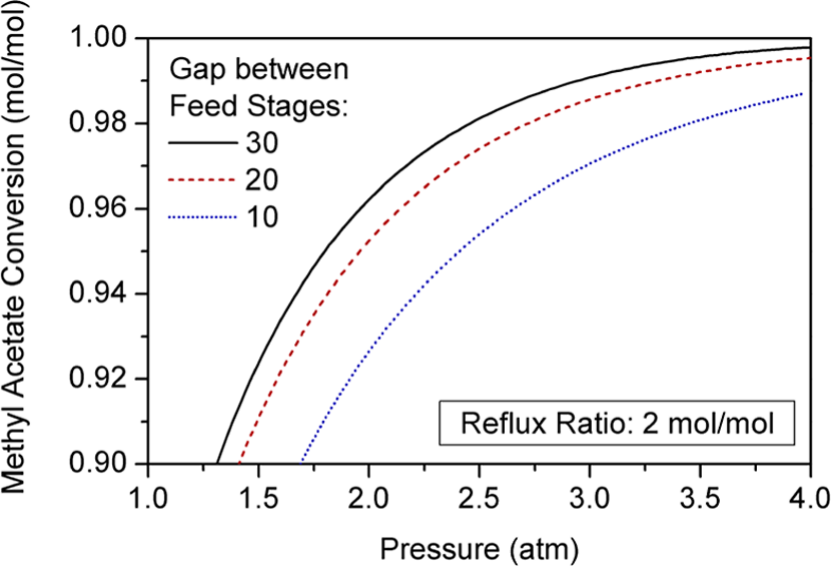
Effect of pressure on methyl acetate conversion.

Nevertheless, as the pressure increases, product generation shifts toward the stages in the lower section of the column. As represented in Figure 13, the formation of BuAc in trays surrounding the alcohol feed stage (stage 35) becomes progressively less relevant as the operating pressure rises. On the contrary, the generation increases in trays ranging from 55 to 60, especially in the stage where MeAc is fed, which also increases the required number of stages in the stripping zone.

**Figure 13:**
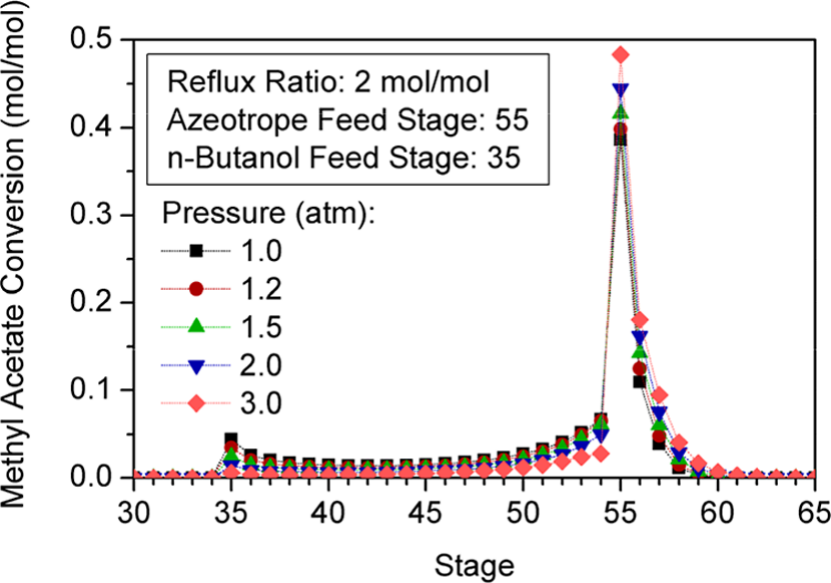
Effect of pressure on the RC reaction profile.

Increasing the pressure diminishes the impact of the number of reactive trays by lowering the conversion rates in some stages, even though these trays contribute to achieving higher overall conversions. This results in a gradual reduction of the difference between conversions for distinct configurations as pressure is raised (Figure 12). In other words, the conversion variation for different reactive trays between feed streams converges as the operating pressure increases. The total conversion line represents the point at which these functions vanish. Taking the effects of this design factor into account, we could theoretically achieve product specifications in both the bottom and overhead product streams could theoretically be possible. However, if the pressure required to reach the necessary conversion (99.93%) to obtain commercial MeOH in the distillate is considered, in practice, only BuAc can be obtained with the desired purity because hot utilities with temperatures higher than high-pressure steam’s would be required.

### 3.2 Prefractionation Column Optimization

The azeotropic composition of MeAc increases at low pressures (Figure 2), i.e., higher amounts of MeOH can be recovered by operating under vacuum conditions in the preconcentration column. The optimal design for this unit is determined by first analyzing how the number of rectifying and stripping stages affects the required reflux ratio and reboiler duty, followed by an evaluation of the total annual cost (TAC) for each configuration, as described in Section 2.2. This TAC can be calculated assuming a 3 year payback period, as shown in eq 3, without including raw materials and RC costs in this analysis.^2^ This brief payback period allows for the swift recovery of the initial investment, reducing the company’s vulnerability to prolonged uncertainties. An investment that pays back its costs in under three years is usually seen as safe and strong, providing a simple way for engineers and managers to decide whether to move forward with projects before doing more detailed financial evaluations.

3 $$TAC=OC+\frac{CI}{3}$$

where OC is the operating cost and CI is the capital investment.

Figure 14 shows that the optimum number of stages for the prefractionation column and the assumed payback period is 26, regardless of the operating pressure. Table 4 details the operating conditions, energy requirements, and associated costs of these designs. Reducing operating pressure to vacuum conditions involves a subtle but gradual increase in capital costs due to the need for a tougher structure for the preconcentrator. Nonetheless, this variation also decreases the energy required in the reboiler, which subsequently results in lower operating expenses. Although reducing the prefractionator pressure further entails higher savings, it also decreases the temperature of the overhead product, which makes unfeasible the use of water as a cold utility considering that its temperature usually ranges from 30 to 45 °C.^24^ Therefore, 0.6 atm can be considered the optimal condition for the PFC. It must be noted, however, that this design involves only a 1.4% savings compared to the same configuration operating at atmospheric pressure. Thereby, the savings may not be high enough for industrial purposes when considering the risks and challenges associated with working under vacuum conditions. Despite this, this study overlooks the limited savings and continues to operate with the vacuum column.

**Figure 14:**
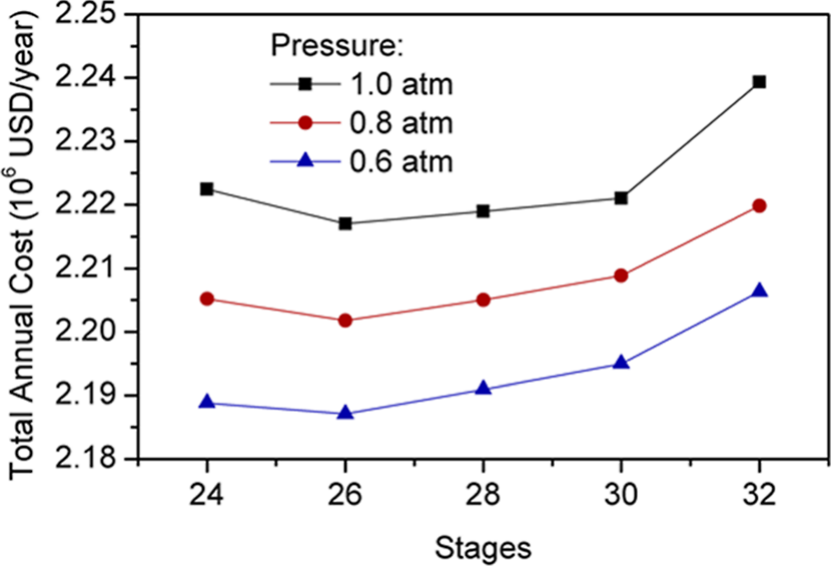
Effect of number of stages and operating pressure on PFC total annual costs.

**Table 4:** Comparison between Prefractionation Columns at Different Pressures

pressure	atm	1.0	0.8	0.6
stages		26	26	26
feed stage		12	12	12
reboiler duty	GJ/t_MeOH_	1.85	1.69	1.55
distillate temperature	°C	54	48	41
CI	×10^6^ USD	3.00	3.01	3.02
OC	×10^6^ USD/year	1.22	1.20	1.18
TAC	×10^6^ USD/year	2.22	2.20	2.19

### 3.3 Reactive Column Optimization

#### 3.3.1 Operating Pressure

The operating pressure of the reactive column is selected by minimizing the reboiler duty required to achieve purity specifications in the bottom stream (Figure 15). The reaction zone of the configurations analyzed ranged from the BuOH feed stage to 10 trays below the MeAc feed stage since the generation of BuAc in this zone is also significant (Figure 13). The minimum energy consumption required by the different configurations is approximately 1.25 GJ/t_BuAc_, and higher pressures are required for configurations with a smaller number of reactive stages, as described in Section 3.1.3.

**Figure 15:**
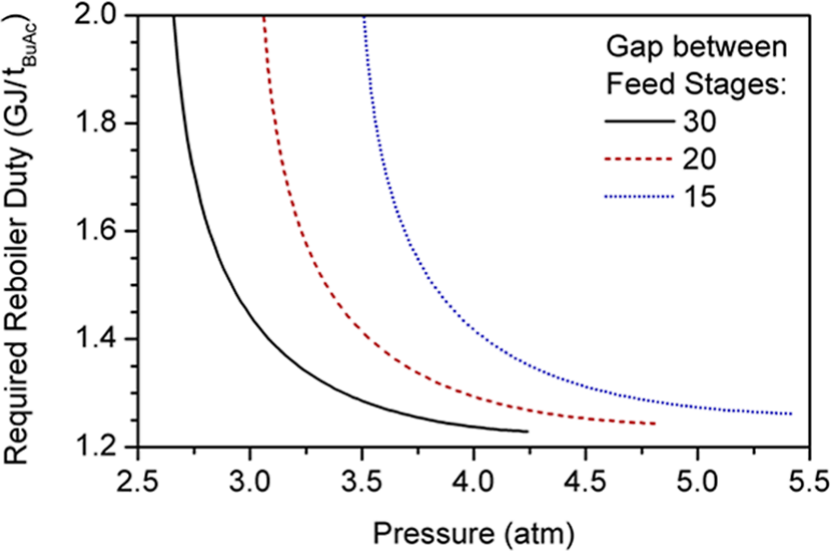
Effect of pressure on required thermal power.

Although operating at higher pressures involves more energy consumption for pressure changers, pump work is negligible compared with reboiler duties (Table 5). If only the energy requirements represented in Figure 15 are considered, then no significant difference can be found between the alternatives. Therefore, capital and operating costs must also be evaluated to determine an optimal design.

**Table 5:** Comparison between RC Designs

stages		48	44	40	38	38
pressure	atm	4.8	5.4	6.8	8.0	9.6
rectifying section stages		9	9	9	9	10
stripping section stages		8	9	10	10	11
reactive stages		31	26	21	19	17
*n*-BuOH feed stage		10	10	10	10	11
azeotrope feed stage		30	25	20	18	17
starting reactive stage		10	10	10	10	11
ending reactive stage		40	35	30	28	27
reactive stage temperature	°C	189	192	204	213	223
bottoms temperature	°C	191	197	208	217	228
required reboiler duty	GJ/t_BuAc_	1.29	1.27	1.31	1.34	1.37
pumps work	MJ/t_BuAc_	2.3	2.6	3.4	4.1	5.0
capital investment	×10^6^ USD	3.64	3.56	3.48	3.44	3.72
operating cost	×10^6^ USD/year	1.37	1.35	1.35	1.35	1.38
total annual cost	×10^6^ USD/year	2.58	2.54	2.51	2.50	2.62

#### 3.3.2 Number of Stages

Rectifying and stripping zones are minimized by analyzing the effect of the number of trays in these sections on the TAC. Figure 16 presents the evaluation for a column with 21 reactive stages. This information indicates that a column with 9 trays in the rectifying zone and 10 stages in the stripping section is appropriate for minimizing costs. The same procedure was followed to determine the optimal stages for different configurations, and similar results were obtained; thus, the height of the RC mainly depends on the size of the reaction section.

**Figure 16:**
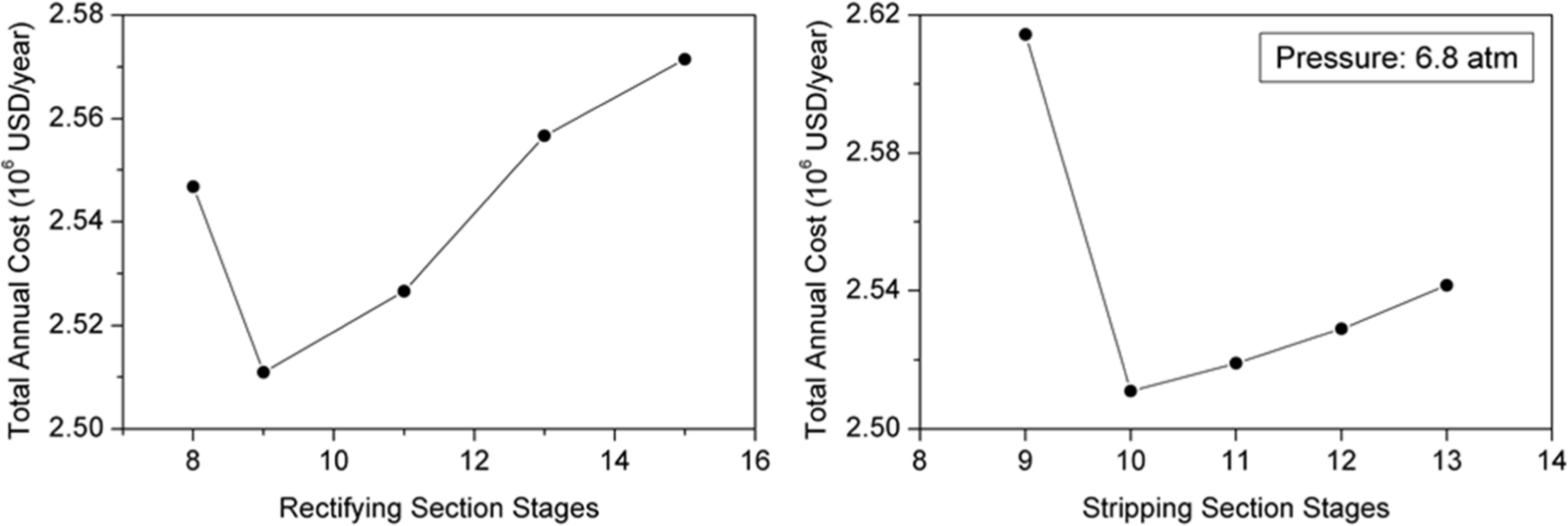
Effect of number of rectifying and stripping section stages on TAC.

Table 5 lists the optimal number of stages, energy consumption, and expenses for the different RC designs. Similar to the PFC, capital investment and operating costs were estimated using the Aspen Process Economic Analyzer tool, which automatically sizes the unit while ignoring raw materials and catalyst costs. Energy-related costs, i.e., operating expenses, are approximately the same for the designs analyzed, although a progressive increase in utility costs is observed for columns that have fewer than 20 reactive stages. Additionally, the minimum number of stages for this process is 38; hence, using fewer reactive stages results in a higher pressure and an increase in equipment costs and capital investment. Therefore, taking the economic data into account, a reactive column comprised of 38 stages and 21 reactive stages is optimal to perform the operation.

### 3.4 Reactive Distillation with a Prefractionation Column (RDPFC)

The separation sequence consists of a prefractionation column (PFC), in which MeOH with the desired composition is collected as a bottom product, and a reactive column (RC), where the MeAc contained in the azeotrope obtained as PFC’s distillate reacts with BuOH to produce BuAc and MeOH (Figure 17).

**Figure 17:**
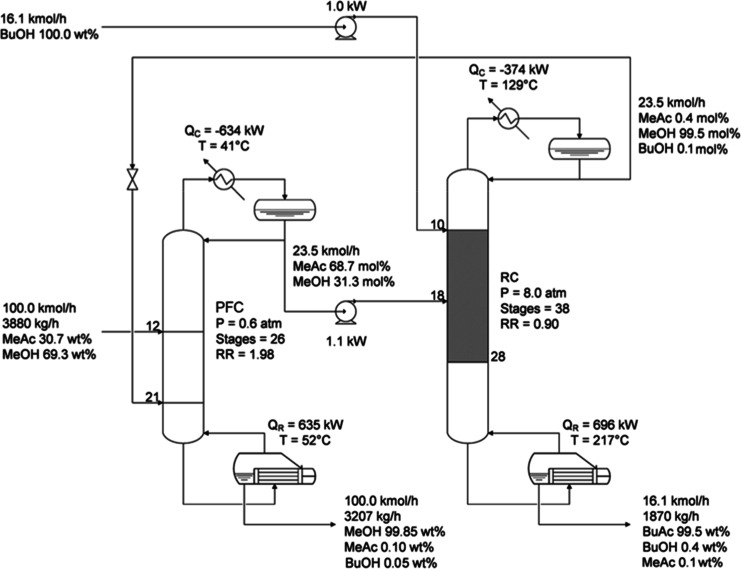
Optimized process flow diagram.

The PFC functions as a preconcentration column that separates feedstock’s excess of MeOH from the azeotropic mixture and also purifies the alcohol recycled from the RC’s overhead product. Due to their difference in composition, MeOH 69.3 mol % in feedstock and MeOH 99.5 mol % in RC’s distillate, the PFC’s feed streams are introduced into stages with matching concentrations in the liquid phase to minimize the energy consumed by the unit. PFC number of stages is such that it complies with the optimal reflux ratio heuristic (RR/RR_min_ = 1.15 in a molar basis).

Figure 18 illustrates the reaction and temperature profiles of the RC. Temperatures reached at reactive stages located between feed stages do not exceed 170 °C; however, temperatures escalate significantly in trays 19–21, reaching 201 °C at the latter. The highest generation of BuAc is achieved in the azeotrope feed stage due to the larger amount of MeAc in this tray (Stage 18 of the composition profile, Figure 19), and stages 18–23 are the most relevant since the combined conversion yielded by these trays is 93% on a molar basis. The overall conversion of reactants is 99.2 mol %.

**Figure 18:**
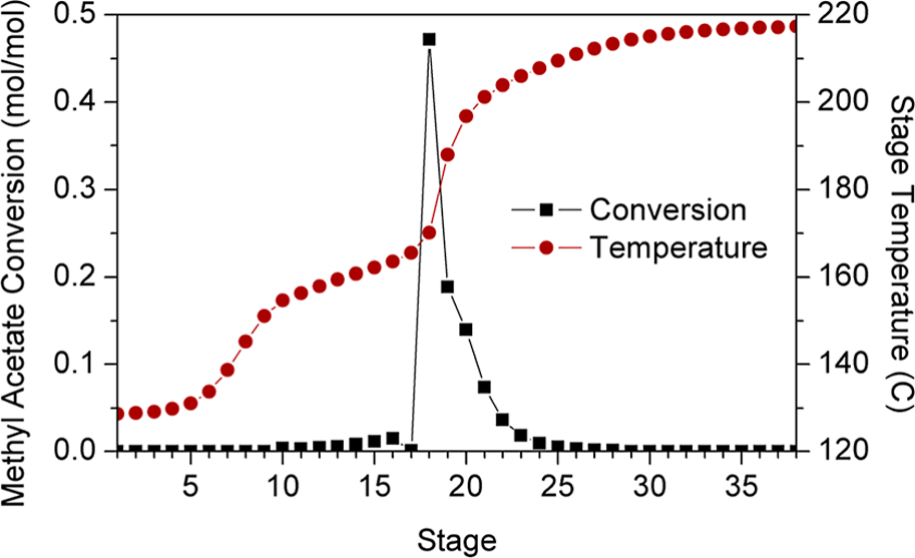
RC temperature and reaction profiles.

**Figure 19:**
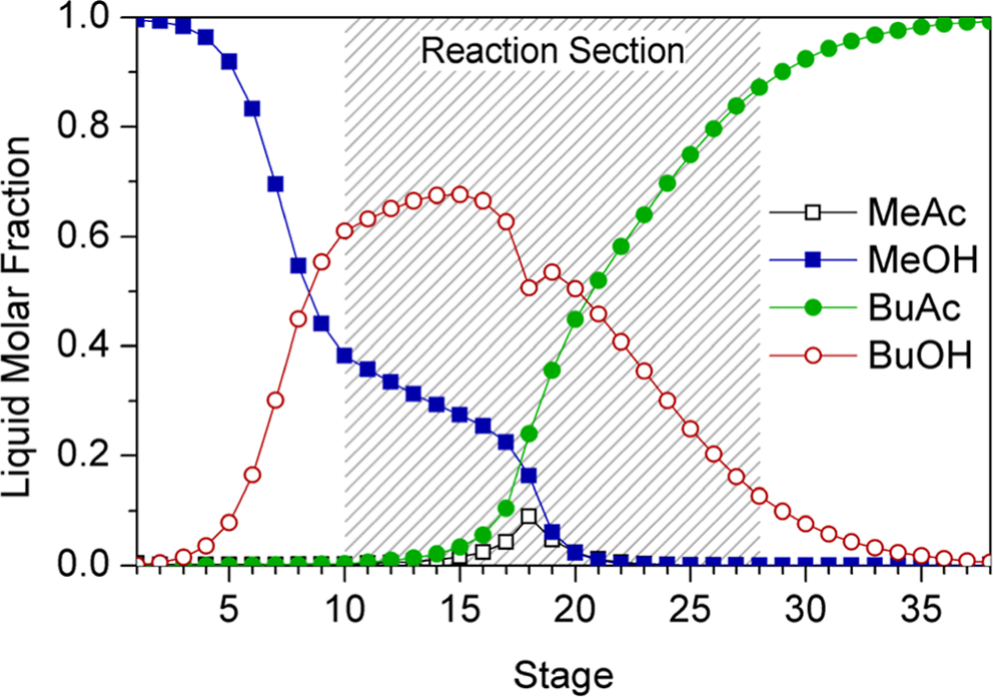
Reactive column composition profile.

#### 3.4.1 Catalyst Limitation

The design of the reactive column is also conditioned by the catalyst’s thermal stability. Temperatures in the reaction zone must not exceed the maximum value recommended for the catalyst to ensure long-term performance while maintaining its activity. As indicated in Section 2, the kinetics used to model the process studied in this document corresponds to the ion-exchange resin Amberlyst 15, whose maximum operating temperature is 120 °C.^34^ Therefore, considering the operating conditions of the optimal design (Table 5), a catalyst with high temperature stability (220 °C) is required, and the catalyst amount will depend on its kinetics and mass-transfer resistance. If a catalyst with lower thermal stability is meant to be used, then a column with a larger number of reactive stages, which operates at lower pressures and thus lower temperatures (Table 5), must be considered. Alternatively, the highest temperature reached in the reaction section, i.e., the last reactive stage, can be reduced by decreasing the column operating pressure. This modification, however, involves a progressive increase of the required reflux ratio, which ultimately entails an increase of the operating costs (Figure 20). A possible catalyst alternative, reaching temperatures up to 220 °C without degradation, may be a calcium oxide catalyst supported on the ZSM-5 zeolites. This catalyst exhibits very high conversions and reactivity in the transesterification of rapeseed oil with methanol.^35^ New kinetic parameters may be required if this catalyst is implemented in our system, which may also affect the optimization.

**Figure 20:**
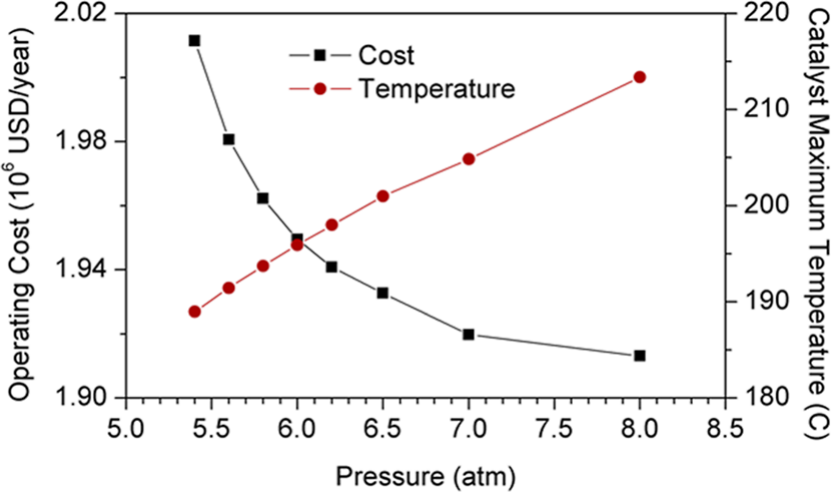
Effect of pressure on operating cost and catalyst maximum operating temperature.

#### 3.4.2 Comparison with Reactive and Extractive Distillation

The performance of the optimized reactive distillation with a prefractionation column (RDPFC) process is benchmarked against the reactive and extractive distillation (RED) technology.^10,23,30^ RED was selected as the primary benchmark because, like the proposed RDPFC system, it is an integrated reactive-separation process, making it the most direct and industrially relevant alternative for a system requiring both transesterification and azeotrope management. The RDPFC and RED comparison is summarized in Table 6. The energy per unit mass of ester required by the reactive column of the RED process is 57% higher than the RC of the RDPFC system due to the use of an entrainer in the unit. Additionally, the energy consumed by the preconcentration column of the RDPFC system is 40% lower than the corresponding column of the RED process. As stated in Section 1.1.1, RED processes require a recovery unit for the entrainer that increases the energy consumption and the TAC of the whole process. The RDPFC technology is more attractive since it can yield products with commercial specifications without the need for an additional unit and reduce the overall energy consumption by 67%. This significant reduction in energy demand is primarily achieved by eliminating the need for an entrainer and its associated recovery column, showcasing the superior efficiency and economic potential of the RDPFC design.

**Table 6:** Comparison between RDPFC and RED Technologies

technology		RDPFC	RED
product purity			
*n*-butyl acetate	wt %	99.50	99.90
methanol	wt %	99.85	99.60
reactive column			
stages		38	43
reactive stages		19	7
operating pressure	atm	8.0	1.0
reboiler duty	GJ/t_BuAc_	1.34	3.14
methanol column			
stages		26	30
operating pressure	atm	0.6	1.0
reboiler duty	GJ/t_BuAc_	1.22	2.04
entrainer column			
stages		N/A	30
operating pressure	atm	N/A	1.0
reboiler duty	GJ/t_BuAc_	N/A	2.65
total energy required	GJ/t_BuAc_	2.56	7.83

## 4 Conclusions

This study analyzes the feasibility and benefits of a reactive distillation with a prefractionation column (RDPFC) process for valorizing the MeAc and MeOH waste from PVA synthesis. The optimized system consists of a 26-stage prefractionation column operating at 0.6 atm and a 38-stage reactive column operating at 8.0 atm, 21 of which are reactive stages where the transesterification reaction occurs. This process achieves an overall MeAc conversion of 99.2 mol %, yielding high-purity BuAc (>99.5 wt %) and methanol (>99.85 wt %) that meet commercial specifications. The primary innovation lies in overcoming multiple azeotropes in a single column. Despite having these mixtures in the same unit, the proposed reactive column is capable of splitting and reacting the mixture, obtaining commercial specifications of BuAc at the bottom and high-purity values of MeOH at the overhead. A prefractionation column is added just to reach the commercial specifications of MeOH, but the RC can separate the components by itself. A comparative analysis highlighted that the RDPFC system requires only 2.56 GJ/t_BuAc_, a 67% reduction in total energy consumption compared to that of the conventional reactive and extractive distillation (RED) process. Eliminating the need for an entrainer and its associated recovery unit primarily achieves this improvement. The study also emphasized the critical role of catalyst thermal stability, indicating that a catalyst capable of withstanding temperatures up to 220 °C is essential for optimal performance. Adjustments to the number of reactive stages and operating pressure will be necessary when using catalysts with lower thermal stability. Ultimately, this research provides evidence that the proposed RDPFC process offers a promising and energy-efficient alternative for recovering valuable products from the PVA residual stream, representing a significant improvement over traditional methods. Future work should address the practical integration of the RDPFC flowsheet into existing PVA plants and investigate the potential impact of trace impurities on reaction kinetics, vapor–liquid equilibrium (VLE), and final product purity, which are critical steps for industrial implementation.

## NOMENCLATURE Symbols

*c*_i_: concentration [kmol m^–3^]
*E*_a_: activation energy [kJ mol^–1^]
*k*: rate constant [m^3^ kmol^–1^ s^–1^ kg_cat_^–1^]
*k*_0_: pre-exponential factor [m^3^ kmol^–1^ s^–1^ kg_cat_^–1^]
*P*: pressure [atm]
*Q*_C_: condenser duty [kW]
*Q*_R_: reboiler duty [kW]
*r*: reaction rate [kmol s^–1^ kg_cat_^–1^]
*R*: universal gas constant [kJ mol^–1^ K^–1^]
RR: reflux ratio [mol/mol]
*T*: temperature [K]

## Abbreviations

CI: capital investment
DWC: divided wall column
BuAc: *n*-butyl acetate
BuOH: *n*-butanol
MeAc: methyl acetate
MeOH: methanol
NRTL: nonrandom two liquid
OC: operating cost
PFC: prefractionation column
PVA: poly­(vinyl alcohol)
RC: reactive column
RED: reactive and extractive distillation
RDPFC: reactive distillation with a prefractionation column
TAC: total annualized costs
